# *Escherichia coli* Frameshift Mutation Rate Depends on the Chromosomal Context but Not on the GATC Content Near the Mutation Site

**DOI:** 10.1371/journal.pone.0033701

**Published:** 2012-03-16

**Authors:** Mariana A. Martina, Elisa M. E. Correa, Carlos E. Argaraña, José L. Barra

**Affiliations:** Centro de Investigaciones en Química Biológica de Córdoba (CIQUIBIC, UNC–CONICET), Departamento de Química Biológica, Facultad de Ciencias Químicas, Universidad Nacional de Córdoba, Córdoba, República Argentina; University of Massachusetts, United States of America

## Abstract

Different studies have suggested that mutation rate varies at different positions in the genome. In this work we analyzed if the chromosomal context and/or the presence of GATC sites can affect the frameshift mutation rate in the *Escherichia coli* genome. We show that in a mismatch repair deficient background, a condition where the mutation rate reflects the fidelity of the DNA polymerization process, the frameshift mutation rate could vary up to four times among different chromosomal contexts. Furthermore, the mismatch repair efficiency could vary up to eight times when compared at different chromosomal locations, indicating that detection and/or repair of frameshift events also depends on the chromosomal context. Also, GATC sequences have been proved to be essential for the correct functioning of the *E. coli* mismatch repair system. Using bacteriophage heteroduplexes molecules it has been shown that GATC influence the mismatch repair efficiency in a distance- and number-dependent manner, being almost nonfunctional when GATC sequences are located at 1 kb or more from the mutation site. Interestingly, we found that in *E. coli* genomic DNA the mismatch repair system can efficiently function even if the nearest GATC sequence is located more than 2 kb away from the mutation site. The results presented in this work show that even though frameshift mutations can be efficiently generated and/or repaired anywhere in the genome, these processes can be modulated by the chromosomal context that surrounds the mutation site.

## Introduction

Replication errors in wild-type cells represent the cumulative effects of several factors including DNA polymerases fidelity, DNA polymerases proofreading and postreplicative mismatch repair [1]–[3]. Replication of *Escherichia coli* chromosome relies mostly on polymerase III (Pol III), responsible for the majority of DNA synthesis, and polymerase I (Pol I), which plays a critical role in lagging strand synthesis [4]. The fidelity of DNA replication depends mainly on the proofreading subunit of Pol III, but it also involves activities of other holoenzyme subunits and the participation of Pol I as well as other accessory DNA polymerases (Pol II, IV, and V). These polymerases possess distinct fidelities, processivities, and catalytic abilities [2].

On the other hand, replication errors are largely corrected by the postreplicative mismatch repair system (MRS), and only a minor fraction results in spontaneous mutations in normally growing cells [3]. The MRS is a highly conserved DNA repair system that greatly contributes to the maintenance of genome stability, increasing the accuracy of DNA replication by 20- to 1000-fold. In *E. coli*, this repair pathway is initiated by binding of MutS to a mismatch. After the recruitment of MutL, this complex activates the strand discriminating endonuclease MutH, which cleaves the newly synthesized, unmethylated daughter strand at the nearest hemimethylated GATC site, and thereby marks it for removal and a repair–synthesis process that involves a variety of other proteins [3].

Using bacteriophage heteroduplexes it was shown that GATC sequences influence the *E. coli* MRS efficiency in a distance- and number-dependent manner from positions both upstream and downstream of a mismatch [1], [5]–[8]. A single hemimethylated GATC sequence was able to direct the repair event to the unmethylated strand. However, over distances in excess of 1 kb the effect of hemimethylated GATC sites on mismatch correction was considerably reduced [1].

Other factors were proposed to affect replication fidelity and evolutionary studies have suggested that mutation rates vary significantly at different positions in the genome. In this sense, genome sequence analysis revealed that in the majority of bacterial species within α- and γ- Proteobacteria genes nearer to the origin of replication had substitution rates lower than genes closer to the replication terminus (although this association was absent in Chlamydiales, and was opposite in Mycobacteria) [9], [10].

The position of an allele relative to the advancing replication fork has also been demonstrated to affect mutation rates. Using different reporter genes located in the two possible orientations relative to the advancing replication fork it was shown, in plasmid as well as in chromosomal DNA, that for some alleles there is a difference in fidelity of replication between the leading and lagging strands [4], [11]. Furthermore, it was also reported a difference in the nucleotide content of the leading and lagging strands of bacterial genomes [12]. However, whole-genome sequencing analysis of *Salmonella typhimurium* mutants, devoid of the major DNA repair systems involved in repairing common spontaneous mutations caused by oxidized and deaminated DNA bases, showed no significant mutational bias with regard to leading and lagging strands or to chromosome position, suggesting that this type of mutations are random in relation to chromosome location [13]. Similarly, the analysis of UV-induced mutations showed that they are produced with similar probability on the leading and the lagging strands during DNA replication [14].

It has also been described a difference in the way that some external mutagen agents affect different chromosomal locations. By whole-genome sequencing of several *E. coli* colonies obtained after chemical mutagenesis, it was observed that the Ori-Ter axis and its orthogonal axis consistently displayed lower mutation density (with up to an order of magnitude difference) [15].

In order to contribute to the identification of factors that influence the accuracy of DNA replication, we investigated whether different genomic contexts and/or a different GATC distribution around a homopolymeric tract are able to affect the rate of chromosomal frameshift mutations in *E. coli*. We found that chromosomal contexts in *E. coli* can affect both, fidelity of the DNA polymerization process and frameshift mismatch repair efficiency. Moreover, we observed that the MRS is able to efficiently repair a chromosomal frameshift mutation even if the nearest GATC site is located more than 2 kb away from the mutation site.

## Materials and Methods

### Bacterial strains and media

*E. coli* wild-type strain (*E. coli* K-12 W3110) and *E. coli mutS* strain in-frame single-gene knockout mutant (JW2703-1), were from the Keio collection (http://ecoli.naist.jp). Strains were grown at 37°C in lysogeny broth (LB) medium with shaking. Media was supplemented with 10 µg/ml streptomycin, 10 µg/ml chloramphenicol or 100 µg/ml rifampicin when indicated.

Restriction enzymes and PCR reactives were from Promega, New England Biolabs or Fermentas. Oligonucleotide primers were from Sigma and sequencing reactions were performed by the University of Chicago Cancer Research Center DNA Sequencing Facility (http://cancer-seqbase.uchicago.edu/).

### Plasmids and strains construction

**Figure 1:**
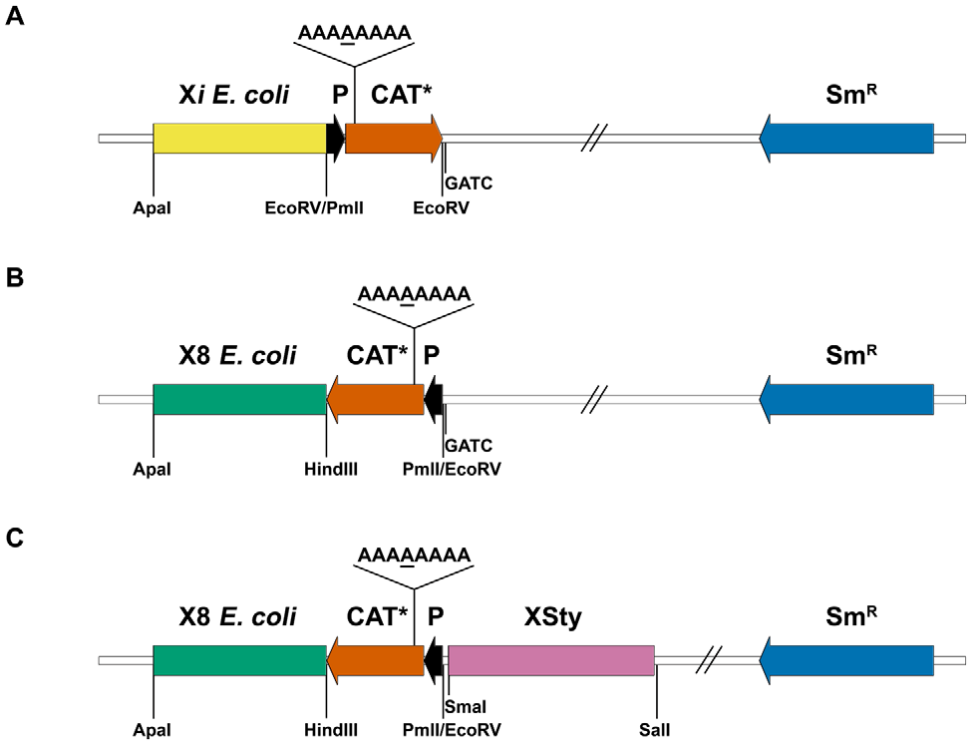
Plasmid constructions. Schematic representation of suicide plasmids used to generate transgenic *E. coli* strains with one mutated copy of the CAT gene situated at different chromosomal locations. A) pKNG derivative plasmids (pKNG-X*i*-P-CAT*) containing an *E. coli* chromosomal DNA fragment (X*i*), a promoter region (P) and a copy of the CAT* coding sequence. B) pKNG derivative plasmid (pKNG-X8-CAT*-P) containing the promoter-CAT* fusion cloned with its transcriptional orientation toward the *E. coli* chromosomal DNA fragment X8. C) pKNG derivative plasmid (pKNG-X8-CAT*-P-XSty) similar to plasmid in “B” but with a *S. typhimurium* chromosomal DNA fragment cloned upstream the promoter-CAT* fusion. See Materials and Methods and Information S1 for a detailed explanation of plasmids and strains construction.

With the purpose of introducing a mutant copy of the chloramphenicol acetyl transferase gen (CAT*) at different locations of the *E. coli* chromosome we generated a series of plasmids containing: a) an *E. coli* chromosomal fragment X*i*, b) an optimized promoter region upstream the CAT coding sequence (P) and c) a mutant copy of the CAT coding sequence (CAT*, Figure 1A). The CAT* gene has an extra adenine nucleotide in a homopolymeric tract (HT) of seven adenines located seven nucleotides downstream the ATG start codon that changes the reading frame of the CAT gene. The three components were cloned into the suicide plasmid vector pKNG which carry a streptomycin resistance cassette (Sm^R^) [16] to obtain plasmids pKNG-X*i*-P-CAT*. We also constructed two other plasmids with the genomic fragment X8 (see Table S2). One of these plasmids is similar to pKNG-X*i*-P-CAT* plasmids but with the Promoter-CAT* fusion cloned with its transcriptional orientation inverted (pKNG-X8-CAT*-P, Figure 1B). In the other plasmid, a *Salmonella typhimurium* chromosomal DNA fragment of 1943 bp containing no GATC sites was cloned next to the CAT* gene of pKNG-X8-CAT*-P to generate pKNG-X8-CAT*-P-XSty (Figure 1C) (see Information S1 and Table S1, S2 for a detailed explanation of plasmids construction).

**Figure 2:**
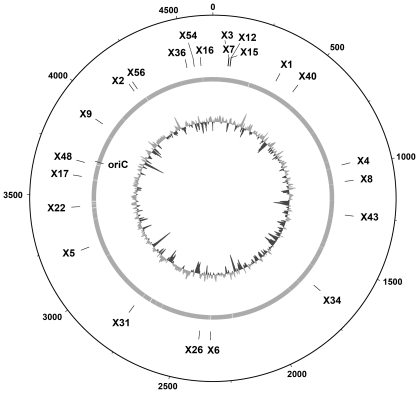
Schematic representation of X*i* fragments position within the *E. coli* chromosome. Base pairs are indicated outside the outer circle (bp×10^3^). The central circle indicates the *E. coli* coding sequences and X*i* denotes the location of the CAT* gene on the different transgenic strains generated in this work. Similar derivative strains were generated in wild-type and *mutS* genetic backgrounds. The inner circle is the genome G+C content. The position of the origin of replication (*oriC*) is shown. See Table S3 for the precise site of CAT* insertion in each strain.

The pKNG derivative plasmids were introduced in wild-type and *mutS E. coli* strains by electroporation, and transgenic strains that incorporated the suicide vector by single homologous recombination between the *E. coli* chromosomal fragment cloned into the plasmid and the corresponding sequence of the chromosome were selected as Sm^R^ colonies. Sm^R^ colonies were randomly picked and the correct insertion of the CAT* gene within the *E. coli* genome was verified by PCR. Twenty-four strains containing the CAT* gene inserted at different locations within the *E. coli* chromosome in a wild-type background and an identical amount of strains in a *mutS* background were obtained. Figure 2 shows the positions were the CAT* gene was introduced within the *E. coli* chromosome.

Chromosomal insertion of plasmids pKNG-X*i*-P-CAT* generated *E. coli* strains in which the nearest GATC site is located between 203 and 929 bp from the mutated site of the CAT* gene (see Table S3). Chromosomal insertion of plasmid pKNG-X8-CAT*-P generated *E. coli* strains in which the nearest GATC site is located 212 bp from the mutated site of the CAT* gene, while plasmid pKNG-X8-CAT*-P-XSty generated strains in which the nearest GATC site is located 2122 bp from the mutated site of the CAT* gene.

### Determination of mutation rates

Mutation rates and 95% confidence intervals (CI) were determined by fluctuation analysis [17]. Strains were analyzed up to 23 times with three to six parallel cultures each time (up to 71 independent cultures in total). Each parallel culture was inoculated with a small number of cells in order to avoid introducing any preexisting mutant and was grown overnight in LB medium containing streptomycin (10 µg/ml). Appropriate dilutions of the overnight cultures were plated onto LB-agar containing streptomycin to determine the total number of viable cells, and aliquots were plated onto LB-agar containing streptomycin (10 µg/ml) and chloramphenicol (10 µg/ml) or rifampicin (100 µg/ml) to determine the number of chloramphenicol resistant (Cm^R^) or rifampicin resistant (Rif^R^) cells, respectively, following incubation overnight at 37°C. The mutation rate was determined from the distribution of the number of mutants in the cultures by the MSS maximum-likelihood method using SALVADOR 2.3 [18]. Fluctuation assays were combined when homogeneous and a new mutation rate was calculated from the combined data according to Rosche and Foster [17].

Mutation rates are given relative to the mutation rate of strain X1, selected at random as the reference strain. The Cm^R^ mutation rate value of Wt-X1 strain was 5.38 (95% CI, 4.93–5.83)×10^−8^ and that of *mutS*-X1 strain was 8.14 (95% CI, 7.74–8.51)×10^−6^. The Rif^R^ mutation rate value of Wt-X1 strain was 5.75 (95% CI, 4.91–6.64)×10^−9^ and that of *mutS*-X1 strain was 7.20 (95% CI, 6.65–7.72)×10^−7^.

Statistical differences were evaluated using the Student's t-test on the relative mutation rate and relative efficiencies values, accepting P = 0.05 as significant.

Restoration of CAT reading frame in Cm^R^ cells was analyzed by DNA sequencing. We selected at random 39 Cm^R^ colonies, amplified by PCR a 321 pb sequence containing the HT (using primers Ps and SCATa, see Table S1) and determined their DNA sequence. All sequences contained a −1A mutation in the HT, reestablishing the wild-type CAT reading frame.

### Correlation analysis

The distance between the site of insertion of the CAT* adenine HT in the genome and the nearest GATC was determined in all derivatives strains from the genome sequence of *E. coli* K12 W3110 (GenBank accession no. gi: AP009048). The total number and density of GATC sites within a 1 kb DNA region around the adenine HT of the CAT* gene in all strains was determined in a similar way. The adenine HT to Ori site shortest distance was also estimated according to the CAT* chromosomal insertion location (Table S3).

The correlation between the mismatch repair efficiency of all strains analyzed and the density of GATC sites, or the HT to Ori site distance, was estimated using the Spearman's rank correlation coefficient. The correlation between the mismatch repair efficiency and the number of GATC sites or the adenine HT to nearest GATC distance was calculated using the Pearson's correlation coefficient on the rank variables instead, due to the existence of ties in one of the variables. The existence of differences in the mismatch repair efficiency as a function of the CAT* orientation relative to the Ori was evaluated using the Kruskal-Wallis test. In all cases, a value of P = 0.01 was accepted as significant.

## Results

### Effect of chromosomal context on frameshift mutation rate

In order to analyze the effect of chromosomal context on the *E. coli* frameshift mutation rate, we constructed a series of isogenic strains containing a mutated copy of the chloramphenicol acetyl transferase (CAT*) gene at different locations in the *E. coli* chromosome (Figure 1 and Figure 2).

In all strains generated with pKNG-X*i*-P-CAT* plasmids, the *E. coli* chromosomal DNA starts at a distance of 191 bp from the mutated site of the CAT* gene (171 bp corresponding to the promoter region, 7 bp corresponding to the coding sequence of the CAT gene until the mutated poly-A tract located 7 bp downstream the ATG and 13 bp corresponding to restriction sites used for cloning) (see Figure 1A). As plasmid DNA is expected to affect the mutation rate similarly in all strains, any observed difference in the frameshift mutation rate will be a consequence of the different chromosomal DNA regions located at least 191 bp away from the mutation site (i.e. we measured a long distance chromosomal context effect).

As the CAT* gene has a frameshift mutation at the beginning of its coding sequence, transgenic strains were chloramphenicol sensitive. Thus, we identified derivatives of the starting strains that restore the CAT reading frame (Cm^R^) by plating the cells on medium containing chloramphenicol. We measured the frameshift mutation rate (−1A) as the Cm^R^ emergence rate by fluctuation analysis. −1A frameshift mutations at the HT were confirmed by DNA sequencing (see Materials and Methods).

**Figure 3:**
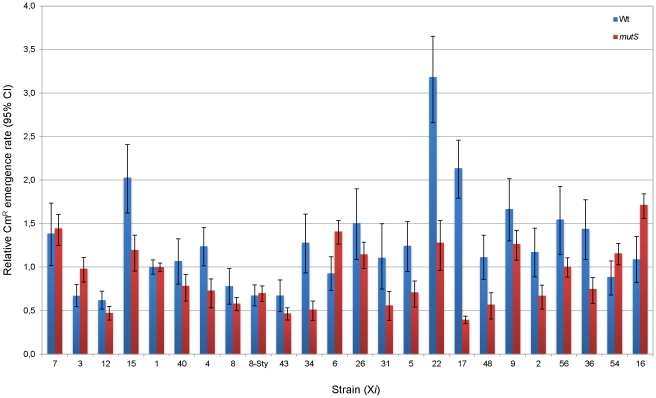
Cm^R^ emergence rates. Cm^R^ emergence rates of wild-type derivative strains relative to Wt-X1 strain (blue bars) and of *mutS* derivative strains relative to *mutS*-X1 strain (red bars). The mutation rate value of Wt-X1 strain was 5.38 (95% CI, 4.93–5.83)×10^−8^ and that of *mutS*-X1 strain was 8.14 (95% CI, 7.74–8.51)×10^−6^. Error bars indicate the 95% confidence limits on the mutation rates.

In wild-type strain derivatives, we observed a variation of up to 5.1-fold in the Cm^R^ emergence rate among strains with the CAT* insertion in different locations of their genomes, being strains Wt-X12 and Wt-X22 the lowest and the highest mutator strains, respectively (Figure 3, blue bars).

The chromosomal context-dependence of frameshift mutations in wild-type derivatives could indicate a context-dependence of the fidelity of the DNA polymerization process, a context-dependence of the frameshift mismatch repair efficiency, or both. In order to discriminate among these possibilities we determined the Cm^R^ emergence rate of isogenic strains generated in a *mutS* background. These strains are deficient in the MRS, and therefore the mutation rate directly reflects the rate of DNA replication errors. As expected, in the *mutS* background the Cm^R^ emergence rate increased in all strains respect to wild-type strains (119 times in average). Strains *mutS*-X17 and *mutS*-X16 represented the lowest and highest mutator strains respectively, with a 4.3-fold difference between them (Figure 3, red bars).

**Figure 4:**
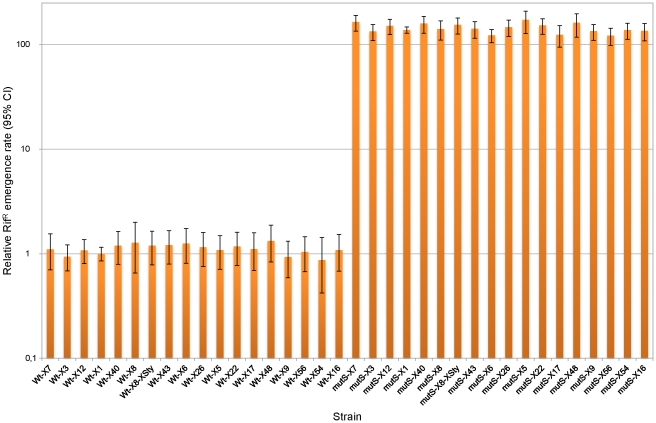
Rif^R^ emergence rates. Rif^R^ emergence rate of some wild-type (Wt) and their corresponding *mutS* derivative strains, relative to Wt-X1 strain. The mutation rate value of Wt-X1 strain was 5.75 (95% CI, 4.91–6.64)×10^−9^. Error bars indicate the 95% confidence limits on the mutation rates.

To rule out that the differences in Cm^R^ emergence rate among wild-type and among *mutS* derivatives strains were due to stochastic mutation rate differences between individual strains or to a possible damage or altered expression of gene(s) due to the random insertion of the reporter CAT* gene within the genome, we determined the mutation rate of an endogenous gene (*rpoB*) located in its natural chromosomal location through the rifampicin resistance (Rif^R^) emergence rate. This analysis showed that those strains that had displayed the maximum differences in Cm^R^ emergence rate did not present statistically significant differences in their Rif^R^ emergence rate, neither in a wild-type nor in a *mutS* background (P>0.05) (Figure 4). Thus, Cm^R^ emergence rate differences observed among wild-type and among *mutS* (Figure 3) derivatives strains could be attributed to a chromosomal context effect on CAT* frameshift mutation rate.

**Figure 5:**
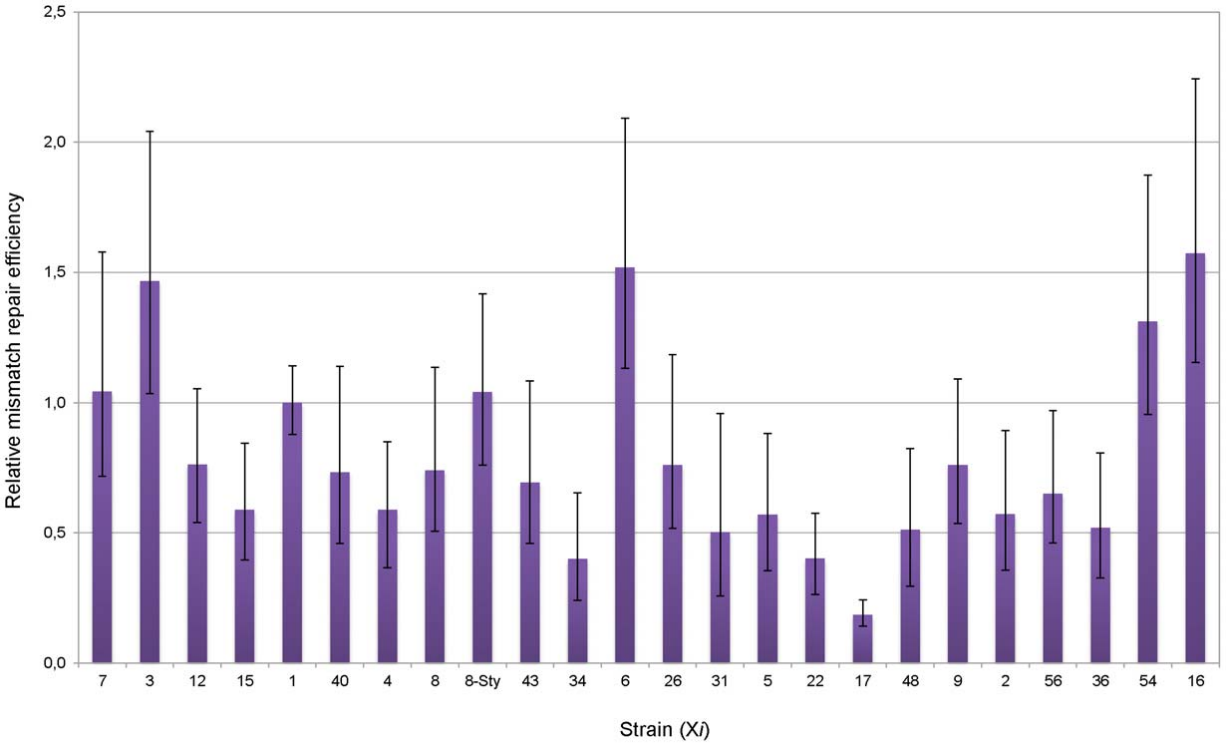
Relative mismatch repair efficiency. The mismatch repair efficiency was calculated from Figure 3 data as the *mutS*/wild-type Cm^R^ emergence rates ratio.

The fact that the difference in Cm^R^ emergence rate observed among some wild-type derivatives is lost when measured in a *mutS* background (e.g. strains X6 and X22), but that some other strains still display differences (e.g. strains X16 and X17) (Figure 3), indicates that the chromosomal context can affect the efficiency of DNA mismatch repair as well as the fidelity of the polymerization process. The effect of chromosomal context on frameshift mismatch repair was better visualized when the mismatch repair efficiency was analyzed as the *mutS*/wild-type Cm^R^ emergence rates ratio. This analysis revealed that strain X17 has the smallest relative repair efficiency [0.18 (95% CI, 0.14–0.24)] while strain X16 has the highest relative repair efficiency [1.57 (95% CI, 1.15–2.24)]. This represents an 8.5-fold statistical difference (Figure 5, P<0.05). As control, these strains did not present a statistical difference in their *mutS*/wild-type Rif^R^ emergence rate ratio (P>0.05). This result clearly indicates that the chromosomal context is able to affect considerably the frameshift mutation repair efficiency in *E. coli*.

In summary, the analysis of the Cm^R^ emergence rates in *mutS* strain derivatives and that of the mismatch repair efficiency showed that chromosomal contexts can affect both, the fidelity of the DNA polymerization process and the frameshift mismatch repair efficiency. It also showed that while some chromosomal regions affect this processes in one direction, others affect them in the opposite way. For instance, strain X17 has the lowest mutation rate in the *mutS* background (high fidelity of the polymerization process), but one of the highest mutation rates in the wild-type background (low mismatch repair efficiency). In contrast, strain X16 has a high mutation rate in the *mutS* background (low fidelity of the polymerization process), but a low mutation rate in the wild-type background (high mismatch repair efficiency) (Figure 3).

### Effect of transcriptional orientation on frameshift mutation rate

It was reported that HTs could have different frameshift mutation rates depending on the gene orientation relative to the origin of chromosomal replication. Using a series of *lac* alleles in an *E. coli* mismatch repair defective background it was shown that reversion frequencies of (+1) frameshift mutations [addition of G·C to a (G·C)_6_ run or addition of A·T to an (A·T)_6_ run, respectively] and (−1) frameshift mutation [loss of G·C from (G·C)_6_] showed a 1.5- to 5-fold difference between leading and lagging strands of replication. However, the reversion frequency of a (−1) frameshift mutation [loss of A·T from (A·T)_7_] did not show any difference between both strands of replication [11].

**Figure 6:**
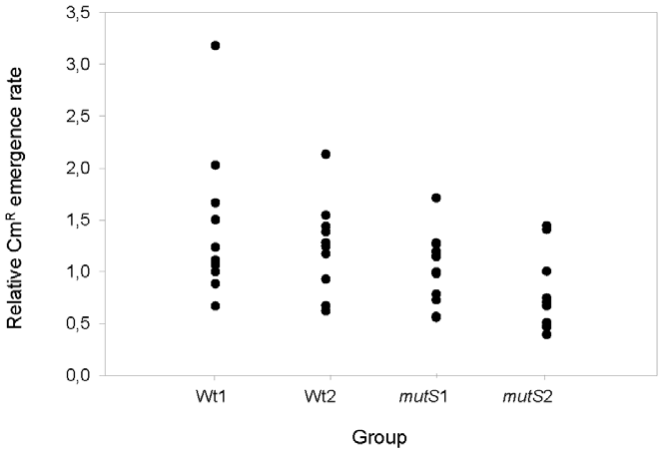
Mutation rates within leading and lagging strands of replication. Cm^R^ emergence rates within the leading and lagging strands of replication in wild-type (Wt1 and Wt2) and *mutS* (*mutS*1 and *mutS*2) backgrounds.

As described before, our CAT* gene contains an adenine insertion (+1) that changes an A_7_ to an A_8_ HT. The transcriptional orientation of the CAT* gene inserted within the *E. coli* chromosome was similar to that of the advancing replication fork in eleven strains (X3, X4, X5, X6, X9, X15, X16, X17, X34, X40, X54) and opposite in the other eleven strains (X1, X2, X7, X12, X22, X26, X31, X36, X43, X48, X56). We found no significant differences between the (−1A) frameshift mutation rate [loss of A·T from (A·T)_8_] of the different copies of the CAT* gene inserted in one or other transcriptional orientation relative to the advancing replication fork on neither, wild-type or *mutS* backgrounds (P>0.05) (Figure 6).

### Effect of GATC density on frameshift mutation

Methylation of adenines in GATC sequences is essential for the correct functioning of the MRS [3], [19]. As mentioned before, using bacteriophage heteroduplexes it was shown that the mismatch repair efficiency falls off with the decrease in the total number of GATC sites and with the increase in the mismatch to GATC distance [1], [5]–[8]. Moreover, in excess of 1 kb the effect of hemimethylated GATC sites on mismatch correction was almost unnoticeable [1].

Considering that our CAT* gene was placed in different chromosomal locations with different densities of GATC sites, we analyzed if there existed a correlation between the frameshift mutation rate determined for the different strains and: 1) the density of GATC sites around the adenine HT, or 2) the distance of the HT to the nearest GATC site.

**Figure 7:**
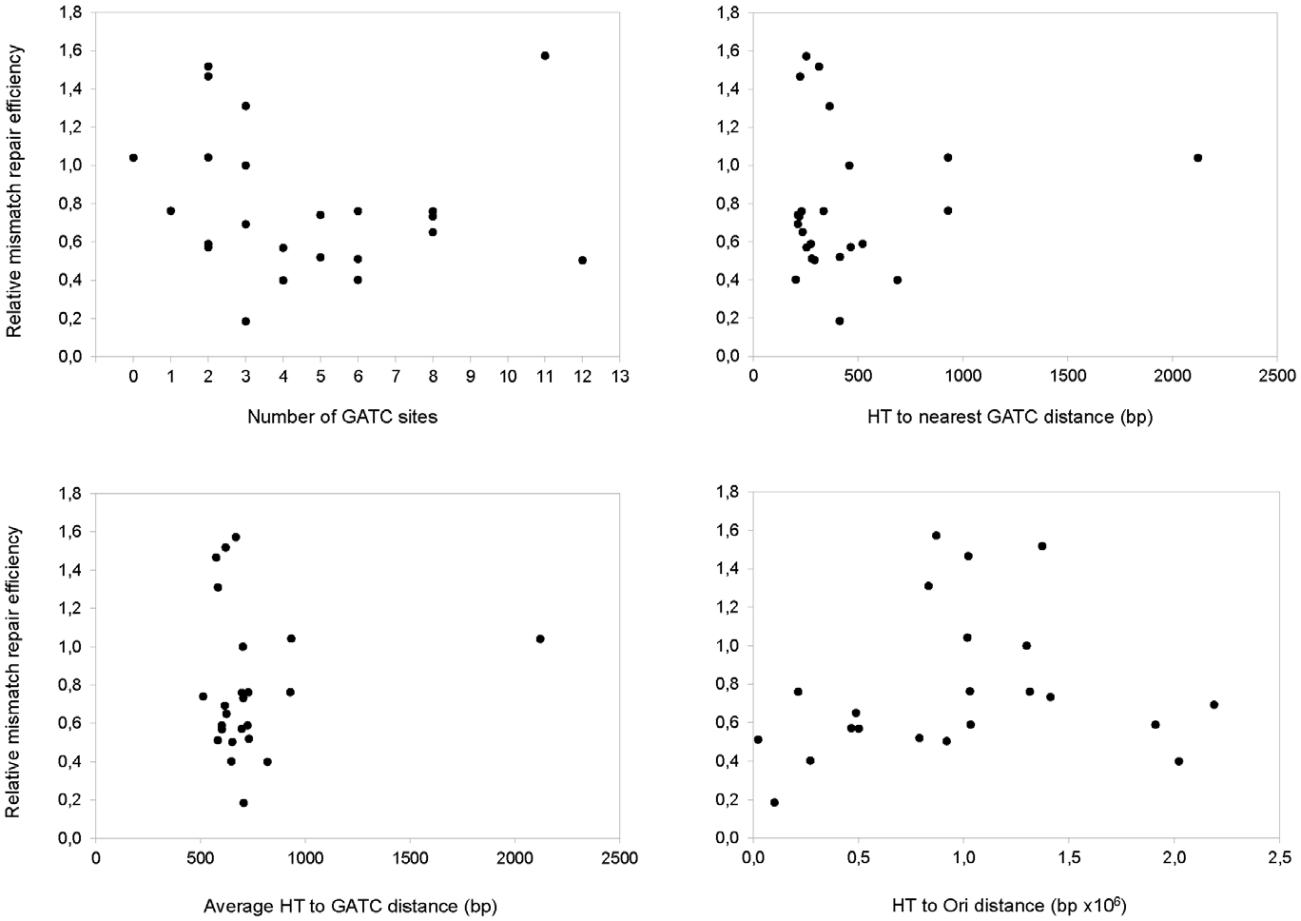
Effect of GATC sites on mismatch repair efficiency. Relative mismatch repair efficiency as a function of A) the number of GATC located within 1 kb flanking the adenine HT, B) the adenine HT to nearest GATC distance, C) the average distance to GATC sites located within 1 kb flanking the adenine HT (plus the nearest GATC site for strain X8-XSty at 2122 pb) and D) the adenine HT to Ori distance.

All constructed strains have one GATC site located immediately after the CAT* coding sequence 929 bp 3′ to the HT (Figure 1A) and GATC sites located at different distances within the chromosomal DNA on the other side (the nearest ranging from 203 to 1713 bp, Table S3). The analysis of all strains generated in this work showed that there is no correlation between the mismatch repair efficiency and the number of GATC sites located within 1 kb flanking the HT of the CAT* gene (P<0.05, Figure 7A). The mismatch repair efficiency did not correlate either with the distance of the HT to the nearest GATC site (P<0.05, Figure 7B), nor with the average distance of the GATC sites within 1 kb of the chromosomal region flanking the HT (P<0.05, Figure 7C) nor with the HT to the Ori site distance (P<0.05, Figure 7D).

In order to maximize the effect of GATC depletion in the flanking regions of the HT, we generated new *E. coli* strains in which the CAT* gene was flanked on one side by an *E. coli* genomic region depleted of GATC sites, and on the other side by a large genomic DNA fragment from *S. typhimurium*, also lacking GATC sites (X8-XSty, Figure 1C). As a control, the CAT* gene was placed in the same *E. coli* chromosomal location without the genomic DNA fragment from *S. typhimurium* (X8, Figure 1B). These new strains have GATC sites located at considerably different distance from the HT. While strains Wt-X8 and *mutS*-X8 have the nearest GATC site located 212 bp from the HT, strains Wt-X8-XSty and *mutS*-X8-XSty have the nearest GATC site located 2122 bp from the adenine HT of the CAT* gene. The Cm^R^ emergence rate of *mutS*-X8-XSty strain was similar to the mutation rate obtained for strain *mutS*-X8 (Figure 3), indicating a similar error rate on the polymerization process. Surprisingly, the Cm^R^ emergence rate of Wt-X8-XSty strain was also similar to the mutation rate obtained for strain Wt-X8 (Figure 3), indicating that there is no difference in the MRS efficiency either. These results contrast considerably with those obtained using bacteriophage heteroduplex DNA, where a considerable decrease of the mismatch repair efficiency was observed as the GATC to mismatch distance increased or as the amount of GATC sites decreased [1], [5]–[8]. Our results showed that within the *E. coli* chromosomal DNA the mismatch repair system is able to normally repair a frameshift mutation even when the nearest GATC site is located more than 2 kb away from the mutation site.

## Discussion

It is well known that the mismatch repair efficiency depends not only on the nature of the mismatch but also on the base sequence on its vicinity [20]–[22]. However, little is known about long range effects of chromosomal sequences on the fidelity of the DNA polymerization process and on mismatch repair efficiency.

In this study we examined the long distance effect of chromosomal context on *E. coli* frameshift mutation rate. We inserted a reporter gene containing a frameshift mutation (+1A) at different locations in the *E. coli* chromosome and analyzed the effect of chromosomal context on the rate of mutations, restoring the normal reading frame of the reporter gene, at each chromosomal location. We showed that some chromosomal regions exert a context-dependent effect on HT frameshift mutation rate (up to five-fold difference) in an *E. coli* wild-type background. If this effect reflected only the context-dependence of mismatch repair efficiency, the mutation rate differences among chromosomal regions should be lost in cells lacking a functional DNA mismatch repair system. However, we found that while in a MRS deficient (*mutS*) background some strains lost their mutation rates differences, some others retain statistically significant differences (up to four-fold). Thus, while for some chromosomal regions the context-dependence of HT mutation rate reflects the efficiency of DNA mismatch repair, for others it reflects a context effect on the fidelity of the polymerization process or an effect on both processes. In this sense, the analysis of the DNA mismatch repair efficiency (*mutS*/wild-type Cm^R^ emergence rates ratio) showed that frameshift repair efficiency could vary more than eight-fold when the effect of different chromosomal regions was compared. Thus, we show that chromosomal sequences located more than 190 bp away from a HT are able to affect both, frameshift generation and repair.

A chromosomal context effect on mutation rates was described in *Saccharomyces cerevisiae* [23]. A 16-fold difference in the rate of frameshift mutations was observed between isogenic wild-type yeast strains with a reporter gene placed at different locations in the genome. As the mutation rates among mismatch repair deficient derivatives were substantially reduced, it was suggested that the variation in efficiency of DNA mismatch repair in different locations of the genome probably reflected some aspect of chromosome structure [23]. Although the structure of the bacterial chromosome is very different from the eukaryotic chromosomal structure, something similar could be happening in bacteria. We do not know yet if, for example, any nucleoid-associated protein could be participating in the chromosomal context effect on the fidelity of DNA replication and/or on the mismatch repair efficiency that we observed in *E. coli*.

More recently, another chromosomal context effect on mutation rates was reported in bacteria. It was shown that mismatch repair at a trinucleotide repeat array stimulates instability of a 275-bp tandem repeat located up to 6.3 kb away on the *E. coli* chromosome. These results provide evidences that mismatch repair (via the MRS) at one type of repetitive DNA has the potential to influence the stability of another one, even when they are separated by a long distance [24].

Although some studies have shown a correlation between the mutation rate and the distance of the mutation site to the origin of replication (Ori) [9], [10], not all of them did. The reversion rate of two *lacZ* alleles inserted at four positions in the *Salmonella enterica* chromosome did not show any correlation with the *lacZ*-origin of replication distance. Even more, the mutation rate at an intermediate locus was higher than those at loci nearer to and farther from the replication origin, and this higher reversion rate was not the result of an overall increase in mutation rate produced by the insertion at this location, but rather a regional effect [25]. This is in agreement with our observations since we could not find a correlation between the Cm^R^ emergence rate and the distance Ori-HT.

According to Mercier et al. [26] at the cellular level the chromosome is organized into four structured regions, called macrodomaines, plus two unstructured regions. In macrodomaines, collisions between sequences are high-frequency, while two sequences of two different macrodomaines do not interact. In contrast, the sequences of the unstructured regions are able to interact with adjacent sequences of macrodomaines.

We analyze if the Cm^R^ emergence rates or mismatch repair efficiencies observed in the different strains were associated with the distribution of their corresponding chromosomal fragments in these macrodomains/regions, founding no significant correlation (in wild-type or *mutS* background) (not shown).

As described above, in some experimental systems it was shown that there is a difference in fidelity of replication between the leading and lagging strands [4], [11]. On the other hand, our results showed that the adenine (−1A) frameshift mutation occurs similarly within leading and lagging strands of chromosomal replication in wild-type and in *mutS* backgrounds. However, this was not an unexpected result since it has also been already described that the frameshift mutation rates [loss of A·T from (A·T)_7_] of a mutant *lacZ* allele present in opposite orientations on the bacterial chromosome relative to the advancing replication fork showed no significant differences [11].

One well known and essential factor for the correct functioning of the MRS in *E. coli* is the presence of GATC sites which allow the system to distinguish parental from daughter strands through the transitory hemimethylated state of DNA after replication [19]. The role of GATC sequences in the MRS functioning was widely studied by *in vivo* transfection experiments with heteroduplex DNA of bacteriophages without any GATC sequence, with only one GATC located at different distances relative to the mismatch, or with more than one GATC sequence; and also by *in vitro* experiments [1], [5]–[8]. These experiments showed that the efficiency of the MRS was affected by the GATC to mismatch distance. Although a single GATC sequence was able to direct the correction event to the unmethylated strand, in excess of 1 kb the effect of GATC sites on mismatch correction was almost unnoticeable [1]. In our experimental system the CAT* reporter gene was placed in different chromosomal locations having different GATC sites content, and in one of them the distance to the nearest GATC site considerably exceeded 1 kb from the mismatch site (the homopolymeric track). Nevertheless, we did not find any correlation between the mismatch to GATC distance and the MRS efficiency. In fact, we observed that the MRS was able to properly repair a frameshift mutation even if the nearest GATC site was located more than 2 kb away from the mismatch. This result contrasts with those described before obtained by transfection experiments with heteroduplex DNA of bacteriophages [1], [5]–[8]. Several factors can contribute to this discrepancy. For instance, we analyzed the repair of a mismatch generated *in vivo* by the replication machinery while transfection experiments analyzed the repair of heteroduplexes artificially generated and disconnected from the replication process. Another possible explanation for these differences lays in the different Dam remethylation rate of replicative intermediates in plasmid and genomic DNA. It has been reported that the time elapsed between DNA synthesis and GATC methylation can be very short for plasmid molecules (2–4 s), but that it can take up to 1 min for chromosomal DNA in cells with a doubling time of about 100 min [27], [28]. Considering that the hemimethylated state of GATC sites is essential for the MRS, the fast GATC remethylation in plasmids could rapidly decrease the functionality of GATC sites as the mismatch to GATC sites distance increase. Although our results contrast with those that analyzed the effect of plasmid GATCs on MRS efficiency, they agree with the ones obtained with λ heteroduplex DNA showing that the distance between the MutH incision and the damage site(s) can be thousands of nucleotides [29]. In this sense, the accessibility analysis of different gene-specific fragments to methylation-sensitive restriction enzymes at defined time intervals post-replication, indicated that the half-life of hemimethylated DNA could vary between 0.5 and 4.5 min. Assuming that the migration speed of DNA polymerases *in vivo* is, on average, 1000 bp/s [27] this corresponds to at least 30 kb behind the replication fork that would be momentarily unmethylated. Thus, it seems perfectly conceivable that a chromosomal GATC site located 2 kb away from a mutation site could still be efficiently used as the strand discrimination signal to repair a frameshift.

Single-base frameshifts are among the most frequent classes of mutations resulting from replication errors [30]. The best-known model for nucleotide addition and deletion errors involves strand slippage during replication of repeated sequences [31]. The chromosomal context effect reported in this work could also be the result of a variation on the formation rate of the slippage intermediate structures or on their stability, which would appear as a difference in the fidelity of the DNA polymerization process.

Frameshift mutations of HTs within coding sequences are able to produce gene inactivation through a change in the reading frame in a reversible fashion. Analyses of homopolymeric tracts in coding sequences of 99 prokaryotic genomes showed that poly(A) and poly(T) HTs with 3 to 7 bases are overrepresented in most of these genomes, and are preferentially located at the 5′ end of coding genes. Thus, it was proposed that HTs could represent a general and rapid evolutionary mechanism facilitating adaptation and gene regulation across diverse organisms [32]. The results presented in this work are in agreement with this hypothesis and show that while frameshift mutations can be generated and/or efficiently repaired anywhere in the genome, this processes could be modulated by the chromosomal context that surrounds the mutation site.

## Supporting Information

Information S1 **Detailed explanation of plasmids and strains construction.**
(DOC)

Table S1 **Oligonucleotides used in this work.**
(DOC)

Table S2 **Plasmids used and constructed in this work.**
(DOC)

Table S3 **Cloned genomic fragments characteristics.**
(DOC)
