# Supplementary material for: Escherichia coli Frameshift Mutation Rate Depends on the Chromosomal Context but Not on the GATC Content Near the Mutation Site
Source: PLoS One. 2012 Mar 16;7(3):e33701. doi: 10.1371/journal.pone.0033701 (PMC3306285; doi:10.1371/journal.pone.0033701)
Supplement: Information S1 — Detailed explanation of plasmids and strains construction. (DOC) [file pone.0033701.s001.doc]

## Supporting information S1.

## Detailed explanation of plasmids and strains construction

The suicide vector pKNG101 was used to introduce the CAT* reporter gene within the *Escherichia coli* chromosome (Table S2). This plasmid contains a defective *pir* minus origin of replication (*oriR6K*), the *strAB* genes encoding the streptomycin phospotransferase (Sm^R^) as a positive selection marker and multiple cloning sites. This plasmid only replicates in bacterial hosts supplying in *trans* the π protein encoded by the *pir* gene . We generated pKNG derivative plasmids containing: a) an *E. coli* DNA chromosomal fragment, b) an optimized promoter region upstream the CAT coding sequence, c) a mutant copy of the CAT* coding sequence (Figure 1).

The promoter region used to direct the expression of the CAT coding sequence (P) was identical to the P_L-TET01_, a derivative of the constitutive bacteriophage PL-λ promoter, except that: a HindIII site and a PmlI site replace the first three nucleotides of the PL-sense-AatII primer; the sequence TTTCTCCTCTTTAAT was added 5’ to the PL-anti-EcoRI primer; and mutations T-14-C and T-21-C that enhance the promoter strength were included . For P promoter construction, two sets of oligonucleotides were used (P-1s, P-1a, P-2s and P-2a, Table S1). After annealing oligonucleotides P-1s and P-1a, the resulting duplex DNA (containing HindIII and XhoI protruding compatible ends) was cloned into the corresponding restriction sites of plasmid pBKS (Table S2), to generate plasmid pBKS-P-1 (Table S2). Oligonucleotides P-2s and P-2a were also annealed and the resulting duplex DNA (containing XhoI and KpnI protruding compatible ends) was cloned into the corresponding restriction sites of plasmid pBKS-P-1 to generate plasmid pBKS-P (Table S2).

The coding sequence for the enzyme chloramphenicol acetyl transferase (CAT), conferring chloramphenicol resistance, was PCR amplified from plasmid pBR325 (Table S2) with primers CAT*-s and CAT-a (Table S1). Primer CAT*-s contains an extra adenine residue in the sequence corresponding to a 7 poly-A tract of the coding sequence, thus introducing a frameshift mutation in the amplified gene. The PCR product was cloned into plasmid pGEM-T Easy (Table S2) to generate plasmid pGEM-CAT*. A plasmid pGEM-CAT* containing the 5´ end of the CAT* gene close to the SpeI restriction site, was selected. The SpeI-KpnI restriction fragment from plasmid pBKS-P, carrying the P promoter region, was cloned into the corresponding restriction sites of plasmid pGEM-CAT* to generate plasmid pGEM-P-CAT* (Table S2). Then, the HindIII-EcoRV restriction fragment from plasmid pGEM-P-CAT* was cloned into the corresponding restriction sites of plasmid pBKS to generate plasmid pBKS-P-CAT* (Table S2). Finally the ApaI-SmaI restriction fragment from plasmid pBKS-P-CAT* was cloned into the corresponding restriction sites of plasmid pKNG to generate plasmid pKNG-P-CAT* (Table S2).

Plasmids pKNG-X*i*-P-CAT* (were “*i*” is an arbitrary number used to identified each *E. coli* chromosomal fragment inserted) resulted from integration of random ApaI–EcoRV *E. coli* wild-type chromosomal DNA fragments, of about 800 to 2000 bp in length, into the ApaI-PmlI restriction sites of plasmid pKNG-P-CAT* (Table S2, Figure 1A).

Two of the chromosomal DNA fragments used were PCR amplified with primers X1-s/X1-a and X8-s/X8-a, respectively (Table S1). PCR products were cloned into plasmid pGEM-T Easy and then, the ApaI-HindIII restriction fragments were cloned into the corresponding restriction sites of plasmid pBKS to generate plasmids pBKS-X1 and pBKS-X8, respectively (Table S2). The HindIII-EcoRV restriction fragment from plasmid pGEM-P-CAT* (Table S2), carrying the P-CAT* fusion, was cloned into the corresponding restriction sites of plasmid pBKS-X1 to generate plasmid pBKS-X1-P-CAT* (Table S2). Also, the ApaI-SmaI restriction fragment from plasmid pBKS-X1-P-CAT* was cloned into the corresponding restriction sites of plasmid pKNG to generate plasmid pKNG-X1-P-CAT* (Table S2).

Finally, two plasmids were constructed with the genomic fragment X8. In these plasmids the CAT* gene was cloned with its transcriptional orientation inverted relative to the rest of the constructed strains (see Figure 1). In one of these plasmids, the PmlI-HindIII DNA fragment from plasmid pGEM-P-CAT*, carrying the P-CAT* fusion, was cloned into the HindIII-EcoRV restriction sites of plasmid pBKS-X8 to generate plasmid pBKS-X8-CAT*-P (Table S2). Then, the ApaI-SmaI restriction fragment from plasmid pBKS-X8-CAT*-P was cloned into the corresponding restriction sites of plasmid pKNG to generate plasmid pKNG-X8-CAT*-P (Table S2, Figure 1B). In the other plasmid, a 1943 bp *Salmonella typhimurium* LT2*end*-1 chromosomal DNA fragment (basepairs 3187684 to 3189612 from GenBank accession no. gi: 16763390) containing no GATC sites was cloned next to the CAT* gene. This fragment contains: a small part of CDC 811, coding for a putative outer membrane protein (NP_461943.1); *stdC* gene, coding for a putative fimbrial chaperone (NP_461944.1, similar to *ybgP* gene from *E.coli* K12 MG1655 coding for a predicted periplasmic pilus chaperone); and about half of the *stdB* gene, coding for a putative outer membrane usher protein (NP_461945.1, similar to *yqiG* gene from *E.coli* K12 MG1655 coding for a putative membrane protein). None of these genes is related to MRS functioning. To clone this chromosomal sequence, a PCR fragment was amplified from the genome of *S. typhimurium* LT2*end*-1, using primers StySmaI and Sty (Table S1), and the PCR product was cloned into plasmid pGEM-T Easy to generate plasmid pGEM-XSty (Table S2). Finally, the SmaI-SalI restriction fragment from plasmid pGEM-XSty was cloned into the SmaI-SalI restriction sites of plasmid pKNG-X8-CAT*-P to generate plasmid pKNG-X8-CAT*-P-XSty (Table S2, Figure 1C).

References

1. Kaniga K, Delor I, Cornelis GR (1991) A wide-host-range suicide vector for improving reverse genetics in gram-negative bacteria: inactivation of the *blaA* gene of *Yersinia enterocolitica*. Gene 109: 137-141.

2. Jensen K, Alper H, Fischer C, Stephanopoulos G (2006) Identifying functionally important mutations from phenotypically diverse sequence data. Appl Environ Microbiol 72: 3696-3701.
