# Supplementary material for: Escherichia coli Frameshift Mutation Rate Depends on the Chromosomal Context but Not on the GATC Content Near the Mutation Site
Source: PLoS One. 2012 Mar 16;7(3):e33701. doi: 10.1371/journal.pone.0033701 (PMC3306285; doi:10.1371/journal.pone.0033701)
Supplement: Table S1 — Oligonucleotides used in this work. (DOC) [file pone.0033701.s002.doc]

Table S1**.** Oligonucleotides used in this work.

| **Oligonucleotide name** | **Oligonucleotide sequence** | **Restriction sites added** |
| --- | --- | --- |
| P-1s | AGCTT**CACGTG**GACGTCTAAGAAACCATTATTATCATGACATTAACCTATAAAAATAGGCGTATCACGAGGCCCTTTCGTCTTCAC*C* | HindIII **PmlI** *XhoI* |
| P-1a | *TCGAG*GTGAAGACGAAAGGGCCTCGTGATACGCCTATTTTTATAGGTTAATGTCATGATAATAATGGTTTCTTAGACGTC**CACGTG**A | *XhoI* **PmlI** HindIII |
| P-2s | TCGAGTCCCTATCAGTGATAGAGATTGACATCCCTACCAGTGACAGAGATACTGAGCACATCAGCAGGACGCACTGACCGAATTCATTAAAGAGGAGAAA**GGTAC** | XhoI **KpnI** |
| P-2a | **C**TTCTCCTCTTTAATGAATTCGGTCAGTGCGTCCTGCTGATGTGCTCAGTATCTCTGTCACTGGTAGGGATGTCAATCTCTATCACTGATAGGGAC | **KpnI** XhoI |
| CAT*-s | **GGTACC**ATGGAGAAAAAAAATCACTGGATATACC | **KpnI** |
| CAT-a | **AAGCTT**CAGGAATTCGATATCATATCGTCAATTATTACCTCCAC | **HindIII** EcoRV |
| X1-s | **GGGCCC**ATCAGAGTGCGGAACAGTTACGTAATGCCG | **ApaI** |
| X1-a | **AAGCTT**GCGACAAAATTACGTGC | **HindIII** |
| X8-s | **GGGCCC**ATGACCATGATTACGGATTCACTGG | **ApaI** |
| X8-a | **AAGCTT**GCACCATCGTCTGCTCATCCAT | **HindIII** |
| StySmaI | CG**CCCGGG**ACCAGCACGGTCAGTTTTGTTTTCAG | **SmaI** |
| Sty | CCCTCGAGCCATTTTCTGTCCATGCCGGAGTAC |  |
| Ps | CTGAGCACATCAGCAGGACG |  |
| SCATa | GAACACTATCCCATATCACCAGCTC |  |
