# Supplementary material for: Escherichia coli Frameshift Mutation Rate Depends on the Chromosomal Context but Not on the GATC Content Near the Mutation Site
Source: PLoS One. 2012 Mar 16;7(3):e33701. doi: 10.1371/journal.pone.0033701 (PMC3306285; doi:10.1371/journal.pone.0033701)
Supplement: Table S2 — Plasmids used and constructed in this work. (DOC) [file pone.0033701.s003.doc]

Table S2**.** Plasmids used and constructed in this work.

| **Plasmid name** | **Characteristics** |
| --- | --- |
| pBKS | pBluescriptKS. Amp^R^ (Promega) |
| pBR325 | Cm^R^ (GenBank: L08855) |
| pGEM | pGEM-T Easy cloning vector. Amp^R^ (Promega) |
| pKNG | pKNG101 suicide *E. coli* plasmid. Sac^S^, Sm^R^ |
| pBKS-P-1 | pBKS carrying the DNA fragment generated after annealing of primers P-1s and P-1a, cloned into HindIII and XhoI restriction sites. |
| pBKS-P | pBKS-P-1 in which the DNA fragment generated after annealing of primers P-2s/P-2a was cloned into XhoI and KpnI restriction sites.  P-1 plus P-2 regenerate the promoter region P. |
| pBKS-P-CAT* | pBKS in which the HindIII/EcoRV fragment from pGEM-P-CAT* (carrying the P-CAT* fusion) was cloned into the corresponding restriction sites. |
| pBKS-X1 | pBKS carrying an *E. coli* chromosomal region PCR amplified using primers X1-s and X1-a, cloned into ApaI and HindIII restriction sites. |
| pBKS-X8 | pBKS carrying an *E. coli* chromosomal region PCR amplified using primers X8-s and X8-a, cloned into ApaI and HindIII restriction sites. |
| pBKS-X1-PCAT* | pBKS-X1 in which the HindIII/EcoRV fragment from pGEM-P-CAT* (carrying the P-CAT* fusion) was cloned into the corresponding restriction sites. |
| pBKS-X8-CAT*-P | pBKS-X8 in which the PmlI/HindIII restriction fragment from  pGEM-P-CAT* (carrying the P-CAT* fusion) was cloned into the HindIII/EcoRV restriction sites. |
| pGEM-CAT* | pGEM carrying the PCR amplified CAT* gene using primers CAT*-s and CAT-a. A plasmid having the CAT* with the KpnI restriction site close to the SpeI site of the pGEM plasmid was selected. |
| pGEM-P-CAT* | pGEM-CAT* in which the SpeI/KpnI restriction fragment from plasmid pBKS-P carrying the promoter P was cloned into the corresponding restriction sites. |
| pGEM-XSty | pGEM carrying a *S. typhimurium* chromosomal region PCR amplified using primers StySma and StyXho. |
| pKNG-P-CAT* | pKNG in which the ApaI/SmaI restriction fragment from plasmid pBKS-P-CAT (carrying the P-CAT* fusion) was cloned into the corresponding restriction sites. |
| pKNG-X*i*-P-CAT* | pKNG-P-CAT* in which random  *E. coli* ApaI/EcoRV chromosomal restriction fragments were cloned into the ApaI/PmlI restriction sites. |
| pKNG-X1-P-CAT* | pKNG plasmid in which the ApaI/SmaI restriction fragment from plasmid pBKS-X1-P-CAT* (carrying the X1-P-CAT* fusion) was cloned into the corresponding restriction sites. |
| pKNG-X8-CAT*-P | pKNG plasmid in which the ApaI/SmaI restriction fragment from plasmid pBKS-X8-CAT*-P (carrying the X8-CAT*-P fusion) was cloned into the corresponding restriction sites. |
| pKNG-X8-CAT*-P-XSty | pKNG-X8-CAT*-P in which the SmaI/SalI restriction fragment from plasmid pGEM-XSty (carrying a S. typhimurium chromosomal region) was cloned into the corresponding restriction sites. |
