# Supplementary material for: Escherichia coli Frameshift Mutation Rate Depends on the Chromosomal Context but Not on the GATC Content Near the Mutation Site
Source: PLoS One. 2012 Mar 16;7(3):e33701. doi: 10.1371/journal.pone.0033701 (PMC3306285; doi:10.1371/journal.pone.0033701)
Supplement: Table S3 — Cloned genomic fragments characteristics. (DOC) [file pone.0033701.s004.doc]

Table S3**.** Cloned genomic fragments characteristics.

| **X*i*** | **Fragment position in the genome^a^** | | **Orientation^b^** | **Number of GATC sites^c^** | **Distance to nearest GATC site (bp)** | **Average distance to GATC sites (bp)^d^** |
| --- | --- | --- | --- | --- | --- | --- |
|  | **Start** | **End** |  |  |  |  |
| 7 | 83282 | 84966 | Anti-sense | 2 | 929 | 934 |
| 3 | 84961 | 86580 | Sense | 2 | 223 | 576 |
| 12 | 94713 | 96126 | Anti-sense | 1 | 929 | 929 |
| 15 | 96121 | 97371 | Sense | 2 | 274 | 602 |
| 1 | 364407 | 365529 | Anti-sense | 3 | 458 | 703 |
| 40 | 475774 | 477196 | Sense | 8 | 220 | 705 |
| 4 | 974081 | 975304 | Sense | 2 | 521 | 725 |
| 8 | 1067094 | 1068271 | Sense | 5 | 212 | 513 |
| 8-Sty | 1067094 | 1068271 | Sense | 0 | 2122 | 2122* |
| 43 | 1254679 | 1255260^#^ | Anti-sense | 3 | 212 | 617 |
| 34 | 1686925^#^ | 1687762 | Sense | 4 | 688 | 820 |
| 6 | 2335728 | 2336789 | Sense | 2 | 313 | 621 |
| 26 | 2396782 | 2398985 | Anti-sense | 6 | 335 | 729 |
| 31 | 2791988 | 2793042 | Anti-sense | 12 | 292 | 653 |
| 5 | 3207516 | 3208497 | Sense | 4 | 254 | 603 |
| 22 | 3438735 | 3440627 | Anti-sense | 6 | 203 | 648 |
| 17 | 3607314 | 3608811 | Sense | 3 | 412 | 707 |
| 48 | 3686677 | 3687572 | Anti-sense | 6 | 279 | 584 |
| 9 | 3924069 | 3925418 | Sense | 8 | 230 | 698 |
| 2 | 4178194 | 4179025 | Anti-sense | 2 | 465 | 697 |
| 56 | 4199729 | 4200670 | Anti-sense | 8 | 235 | 624 |
| 36 | 4501370 | 4502264 | Anti-sense | 5 | 413 | 732 |
| 54 | 4542326 | 4544087 | Sense | 3 | 364 | 584 |
| 16 | 4580235 | 4581295 | Sense | 11 | 253 | 670 |

^a^bp according to the genome sequence of *E. coli* K12 W3110 (GenBank accession no. gi: AP009048).

^#^ The precise size of these fragments was not determined. While the position of one extreme of the fragment was precisely determined, the other (^#^) indicates only the end of the partial sequence obtained.

^b^CAT* transcriptional orientation relative to the advancing replication fork.

^c^Located within 1 kb flanking the adenine HT.

^d^Located within 1 kb flanking the adenine HT with exception of * witch indicates the closest GATC site to the HT.
